# Protective effect of *Ginkgo biloba* leaves extract, EGb761, on myocardium injury in ischemia reperfusion rats *via* regulation of TLR-4/NF-κB signaling pathway

**DOI:** 10.18632/oncotarget.21372

**Published:** 2017-09-28

**Authors:** Yuping Tang, Guisheng Zhou, Lijun Yao, Ping Xue, Danhong Yu, Renjie Xu, Wen Shi, Xin Yao, Zhaowei Yan, Jin-Ao Duan

**Affiliations:** ^1^ Shaanxi Collaborative Innovation Center of Chinese Medicinal Resources Industrialization, Shaanxi University of Chinese Medicine, Xianyang, 712083, China; ^2^ Jiangsu Collaborative Innovation Center of Chinese Medicinal Resources Industrialization, Nanjing University of Chinese Medicine, Nanjing, 210023, China; ^3^ Department of Pharmacy, The First Affiliated Hospital of Soochow University, Suzhou, 215006, China; ^4^ Changzhou Institute for Food and Drug Control, Changzhou, 213000, China; ^5^ The Children’s Hospital Affiliated to Soochow University, Suzhou, 215006, China; ^6^ Shaoxing Second Hospital, Shaoxing, 312000, China

**Keywords:** EGb761, myocardial ischemia, TLR-4/NF-κB pathway

## Abstract

Beneficial actions of EGb 761 against ischemia/reperfusion (I/R) injury in lung, brain and renal ischemia have been described. However, the relationship between EGb 761 and signal molecules in myocardial ischemia reperfusion has not been well elucidated. In this study, we investigated the effects and mechanism of EGb 761 preconditioning on anti-myocardial I/R injuries *in vivo*. Meanwhile, their potential anti-oxidative stress and anti-inflammation effect were assessed. Hemodynamic parameters were monitored as left ventricular systolic pressure, LV end-diastolic pressure and maximal rate of increase and decrease of left ventricular pressure (dP/dtmax). The oxidative stress indicators and inflammatory factors were also evaluated. Western blot method was used for analysis of toll-like receptor 4 (TLR4), p-TLR4, nuclear factor-κB (NF-κB), p-NF-κB p65, Bax and Bcl-2 protein expressions. EGb 761 significantly improved cardiac function, decreased levels of creatine kinase, aspartate aminotransferase and lactate dehydrogenase. EGb 761 also restrained the oxidative stress related to myocardial ischemia injury as evidenced by decreased malondialdehyde, superoxide dismutase, catalase, glutathione-peroxidase, glutathione reductase activity. Meanwhile, the inflammatory cascade was inhibited as evidenced by decreased cytokines such as tumor necrosis factor-α, interleukin-6 and interleukin-1β. Our results still showed that EGb 761 pretreatment significantly decrease the level of cleaved Bax, and increase the level of Bcl-2 in rats subjected to I/R injury. Simultaneously, the expressions of myocardial TLR4 and NF-κB were significantly decreased. It can be concluded that EGb 761 pretreatment was protected against myocardium I/R injury by decreasing oxidative stress, repressing inflammatory cascade *in vivo* and inhibiting TLR4/NF-κB pathway.

## INTRODUCTION

With the development of economy and society, the incidence of acute myocardial infarction (AIM) showed an upward trend year by year. World Health Organization (WHO) has predicted that by 2020, AMI would become one of the major causes of human death [1-2]. Although drug clinical trials on myocardial ischemic–reperfusion injury (IRI) have yielded encouraging results, the actual effect of clinical treatment is not satisfactory [3]. Therefore, development of new cardioprotective drugs against myocardial IRI remains the research hotspot.

It is universally acknowledged for us that the factors such as oxygen radicals, calcium overload, etc. could induce the pathological process of myocardial I/R injury. Oxidative stress is usually relative to the increased formation of reactive oxygen species [4, 5]. Oxygen derived free radicals are recognized to play a leading part in the genesis of tissue injury in ischemia and reperfusion of the heart [6-9]. It has been proved of benefit in many numerous researches that after ischemic insult resulting in reducing inflammatory responses during reperfusion [10, 11]. Toll-like receptor (TLR) is the important protein that is involved in the nonspecific immunity. By recognizing the pathogen associated molecular patterns, it could induce the cytokine to play the role of anti-infection [12]. By now, over 10 subtypes of TLR have been found in the human body. The different subtype can recognize the different receptor and all TLR ligands can act as the immune adjuvant. According to the previous researches, TLR-4 could activate many cytokine and promote the formation of active oxygen free radicals [13, 14]. The nuclear factor κB (NF-κB) pathway was involved in the tissue injury and stress reaction, while the myocardial ischemia reperfusion could activate the NF-kB pathway in the further process of oxidative stress and calcium overloaded [15, 16].

Ginkgo biloba which is one of the commonly used Chinese medicinal herbs has been be applied to ameliorate memory loss, and respiratory function for the thousand years in East China [17]. Recently, Ginkgo biloba leaves extracts (EGb 761) are amongst the top-selling phytomedicines in the world [18]. The beneficial pharmacologic effects and biological activities of scavenging blood free radical, anti-inflammation, anti-tumor and cardioprotective properties had been proposed to related with EGb 761 [19, 20]. Clinically, EGb 761 has been promoted for the prescribed cure of dementia, vaso-occlusion, and cochleovestibular disorders in Chinese clinic trails [21]. Beneficial actions of EGb 761 against ischemia/reperfusion injury in lung, brain and renal ischemia have also been described [22-24]. The relationship between EGb 761 and signal molecules in myocardial ischemia reperfusion has not been well elucidated, however.

In this study, the cardioprotective properties of EGb 761 were characterized and the evidence of cardioprotective effects which were partly mediated through TLR-4/NF-κB signaling pathways is provided.

## RESULTS

### UPLC-PDA-TOF/MS fingerprint of EGb 761

**Figure 1:**
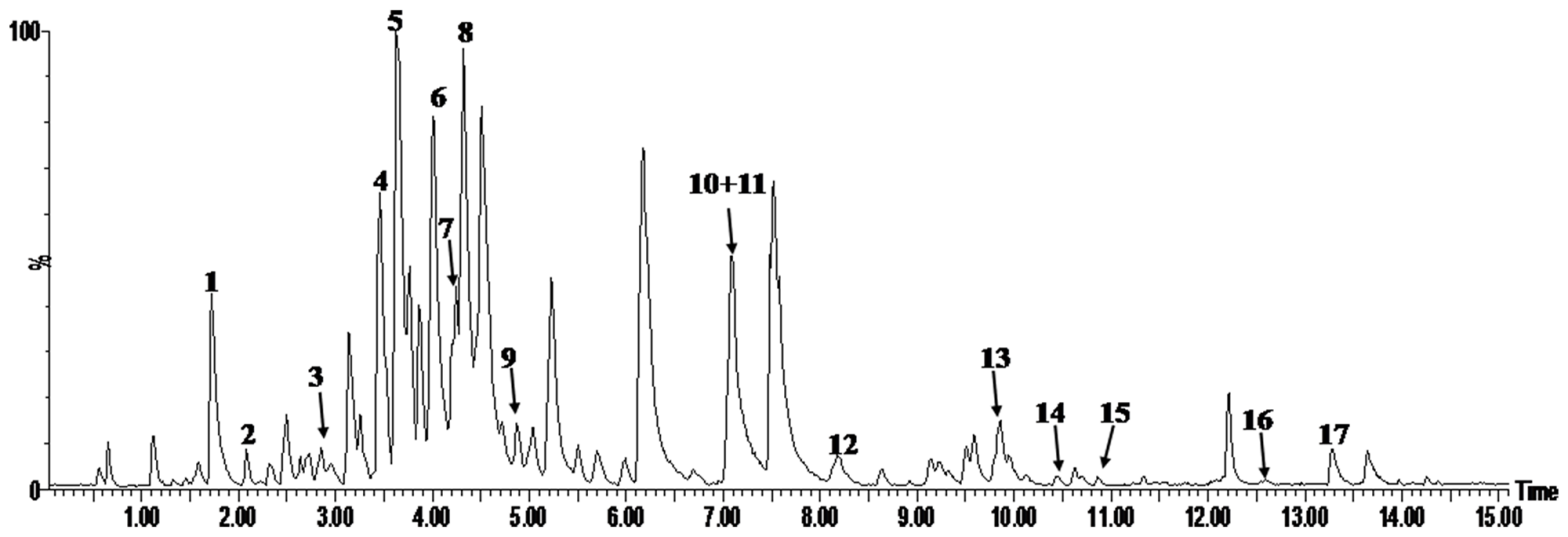
Representative UPLC- PDA -TOFMS chromatograms of EGb 761 in the negative ion mode

UPLC-PDA-TOF/MS analysis was used for the determination of the major constituents of EGb 761. Representative UPLC-MS chromatograms of EGb 761 are shown in Figure 1. The results of analysis demonstrated that the major constituents of EGb 761 were **1** (-)-epigallocatechin (2.21%), **2** (+)-catechin hydrate (0.85%), **3** (-)-epicatechin (1.01%), **4** quercetin 3-*O*-[6-*O*-(ɑ-L-rhamnosyl)-β-D-glucoside] (3.45%), **5** quercetin 3-*O*-β-D-glucoside (6.44%), **6** bilobalide (5.44%), **7** quercetin 3-*O*-[4-*O*-(ɑ-L-rhamnosyl)-β-D-glucoside] (3.21%), **8** ginkgolide C (5.98%), **9** quercetin 3-*O*-ɑ-L-rhamnoside (4.32%), **10** ginkgolide B (1.01%), **11** ginkgolide A (3.99%), **12** luteolin (1.01%), **13** apigenin (2.01%), **14** kaempferol (0.57%), **15** isorhamnetin (0.41%), **16** bilobetin (0.20%), and **17** genkwanin (0.74%).

### Effect of EGb 761 on cardiac function

**Figure 2:**
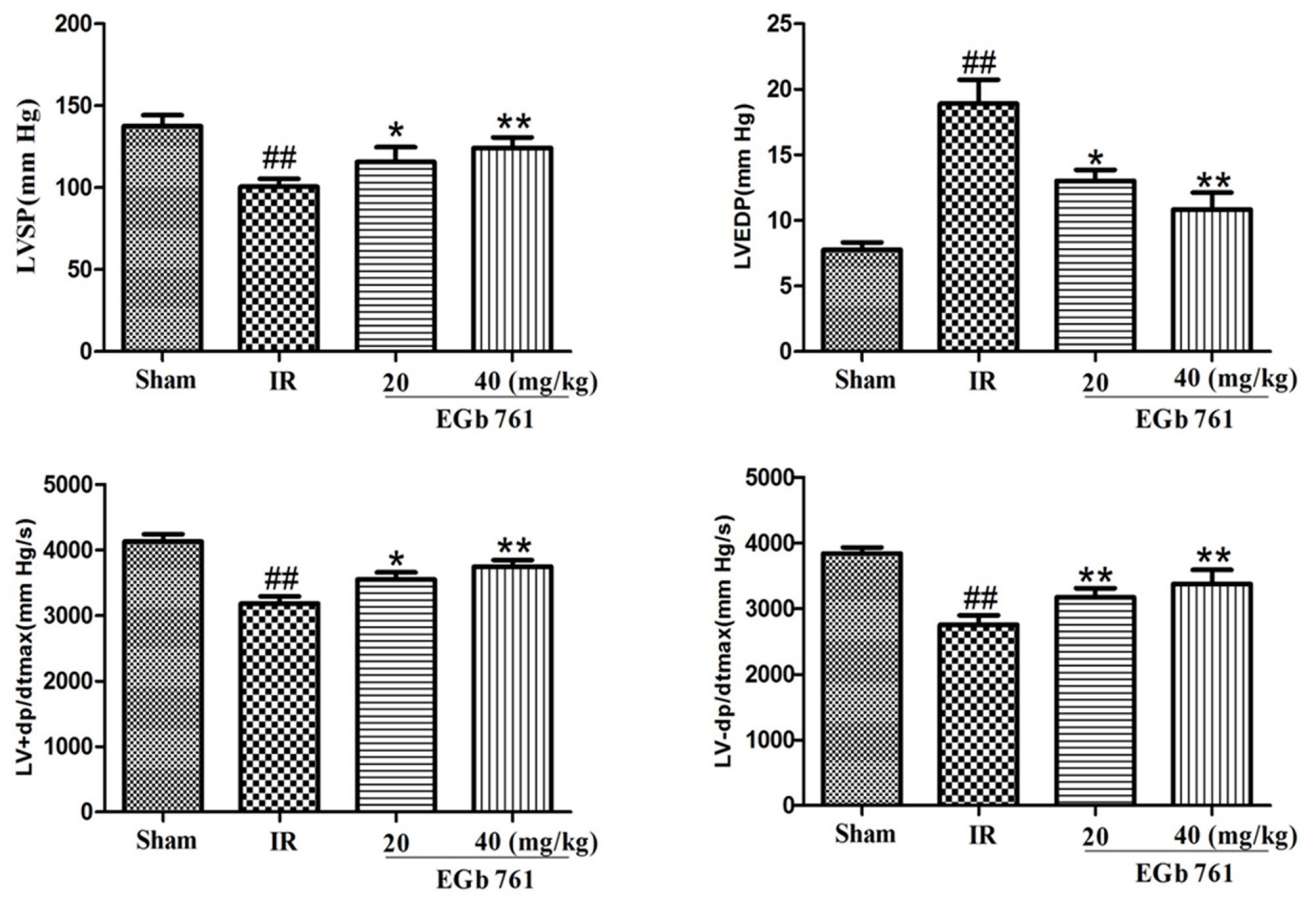
Pre-treatment with EGb 761 improve cardiac contractibility, attenuate hypercontraction and reduce diastolic pressure Values were expressed as means ± SDs. ## P <0.01 compared with sham group. *P < 0.05 and ** P < 0.01 compared with IR group (n=10).

Pre-treatment with EGb 761 in significantly increased LVSP, +dP/dt_ma x_ and -dp/dt_max_, and reduced the LVEDP compared to IR group. These results suggest that structure restoration in the ischemic heart (Figure 2) was translated into cardiac function improvements. Thus, EGb 761 could improve cardiac contractibility, attenuate hypercontraction and reduce diastolic pressure, which is believed to be a major reason for increased O_2_ demand.

### Effect of EGb 761 on the myocardium antioxidant enzymes

**Figure 3:**
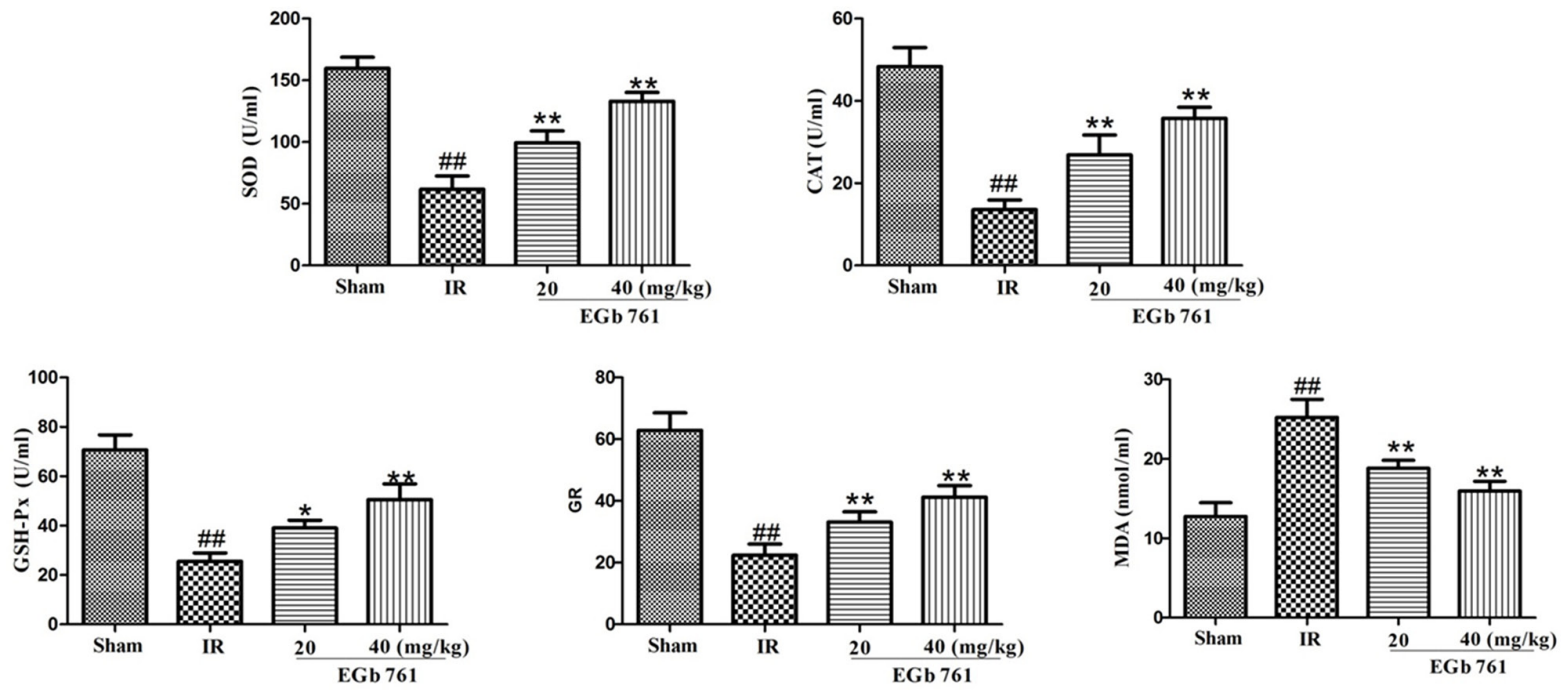
EGb 761 enhanced the antioxidant enzymes activities Values were expressed as means ± SDs. ## P <0.01 compared with sham group. *P < 0.05 and ** P < 0.01 compared with IR group (n=10).

The myocardium antioxidant enzymes (Figure 3) demonstrated insignificant changes following the treatment of EGb 761. Compared with the sham group, IR rats demonstrated significant increase in MDA content, while that of antioxidant enzyme activities, such as SOD, CAT, GSH-Px and GR, were significantly decreased. Treatment of IR rats with EGb 761 enhanced the antioxidant enzymes activities.

### Effect of EGb 761 on release of AST, LDH, CK-MB

**Figure 4:**
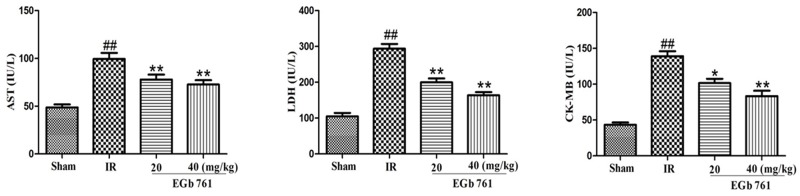
EGb 761 decreased the level of AST, CK-MB and LDH Values were expressed as means ± SDs. ## P <0.01 compared with sham group. *P < 0.05 and ** P < 0.01 compared with IR group (n=10).

Results presented in Figure 4 Indicated that in IR group, the content levels of LDH, CK-MB and AST were remarkably higher than those in the sham group. In comparison with IR group, the levels of AST, CK-MB and LDH contents in the rats treated with EGb 761 were decreased.

### Effect of EGb 761 on Na^+^–K^+^–ATPase and Ca^2+^–ATPase activities

**Figure 5:**
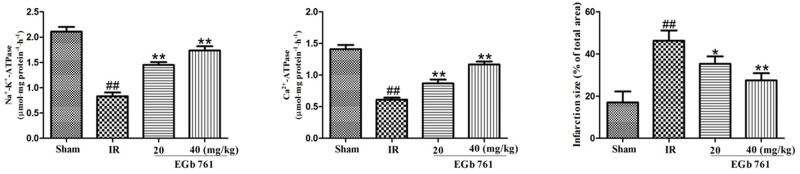
EGb 761 increased Na+–K+–ATPase and Ca2+–ATPase activities and percentage of infarct size Values were expressed as means ± SDs. ## P <0.01 compared with sham group. *P < 0.05 and ** P < 0.01 compared with IR group (n=10).

Results presented in Figure 5 showed us that in IR group the Na^+^–K^+^–ATPase and Ca^2+^–ATPase activities were remarkably lower compared with those in the sham group. Also, compared with IR group, the Na^+^–K^+^–ATPase and Ca^2+^–ATPase activities went up obviously in the rats treated with EGb 761.

### Effect of EGb 761 on infarct size

As shown in Figure 5, the infarct size percentage in IR group was dramatically much higher than the sham group. The EGb 761 group with the administration of 20 or 40 mg/kg could decreased the percentage of infarct size significantly.

### Effects of EGb 761 on pro-inflammatory cytokines

**Figure 6:**
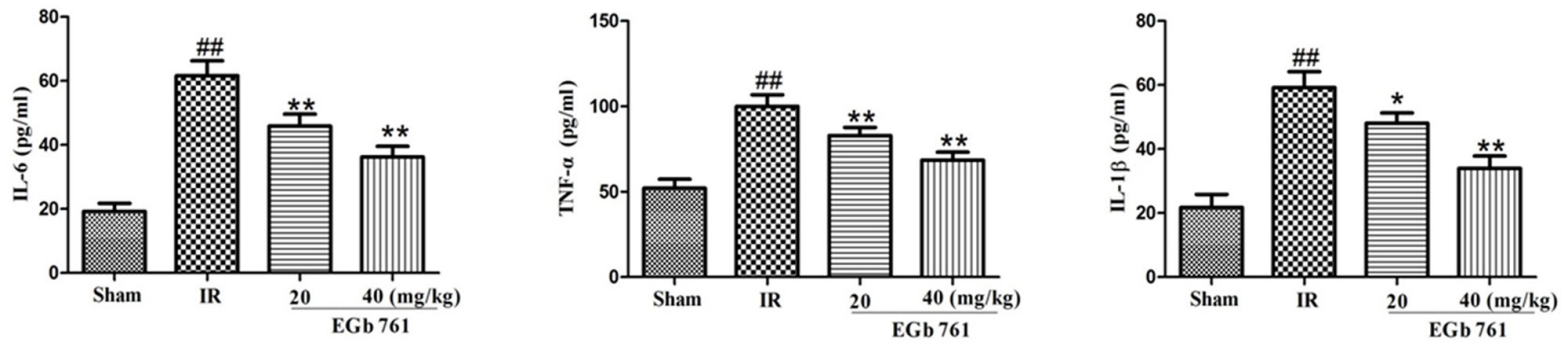
EGb 761 inhibited inflammatory cytokines Values were expressed as means ± SDs. ## P <0.01 compared with sham group. *P < 0.05 and ** P < 0.01 compared with IR group (n=10).

As shown in Figure 6, compared with the sham group, the levels of TNF-α, IL-6 and IL-1β in serum increased significantly in the IR rats group. However, the administrations of EGb 761 (20 and 40 mg/kg) had decreased levels of TNF-α, IL-6 and IL-1β.

### Effect of EGb 761 on myocardial histology

**Figure 7:**
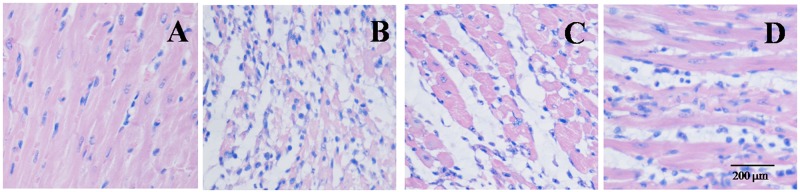
EGb 761 inhibited infiltration of inflammatory cells in Myocardial Histology **(A)** sham group; **(B)** IR group; **(C)** IR + EGb 761 (20 mg/kg) group; **(D)** IR + EGb 761 (40 mg/kg) group. Scar bar: 200 μM (upper panel).

Hematoxylin and eosin staining was adopted in the evaluating the protective effects of EGb 761. Some morphological damage were found in the MI/R group. However, EGb 761 with an administration of 20 and 40 mg/kg weakened the aforementioned morphological disorders (Figure 7).

### Effect of EGb 761 on Bax and Bcl-2 expressions

**Figure 8:**
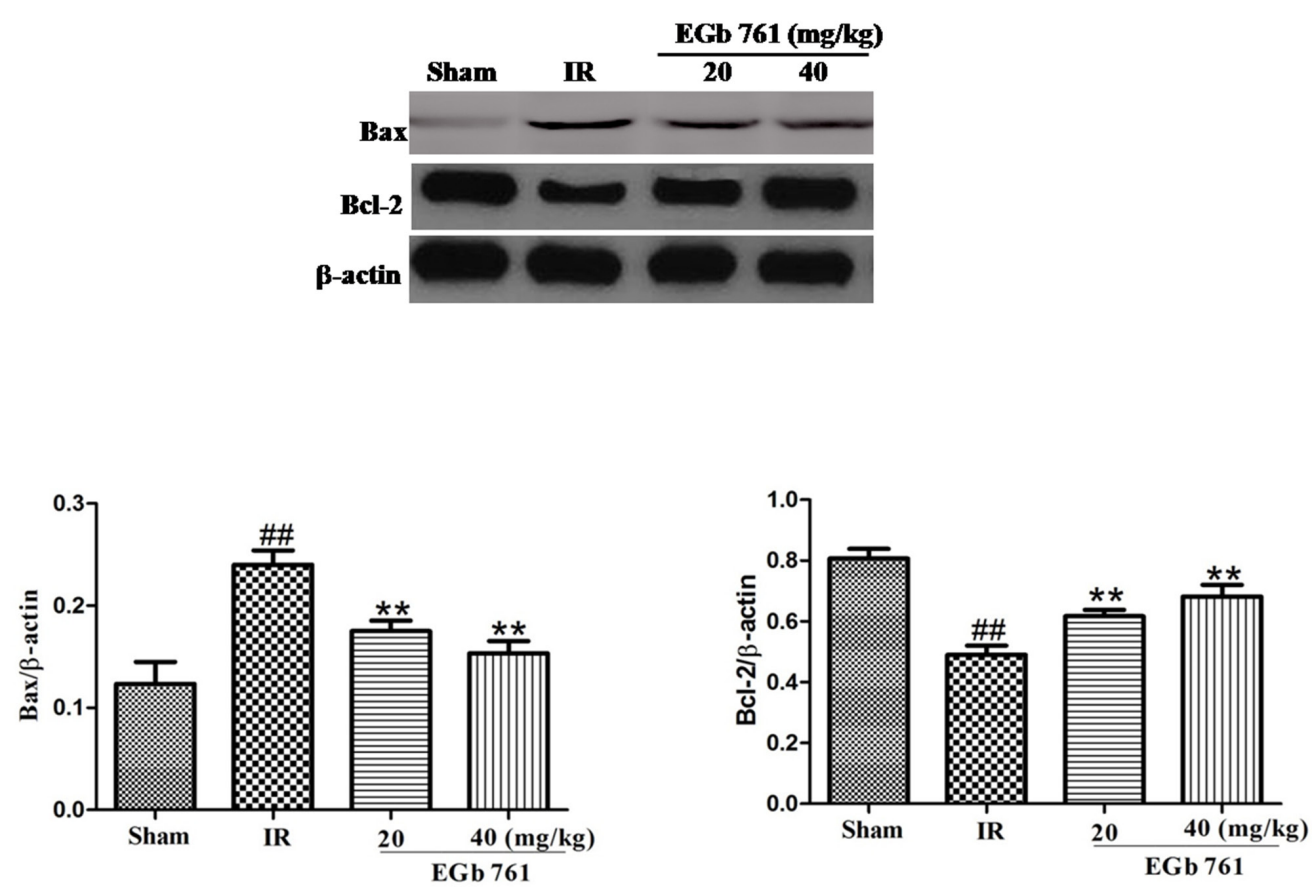
EGb 761 decreased Bax and increased Bcl-2 expressions Values were expressed as means ± SDs. ## P <0.01 compared with sham group. *P < 0.05 and ** P < 0.01 compared with IR group (n=10).

The expression of Bax protein notably increased in the myocardium tissue of I/R group. The expression of Bcl-2 protein obviously decreased by the contrast. The exchanges of MI/R in the Bax and Bcl-2 expressions were correspondingly induced by EGb 761 (20 and 40 mg/kg) administration (Figure 8).

### Effect of EGb 761 on TLR-4/NF-κB signaling pathway

**Figure 9:**
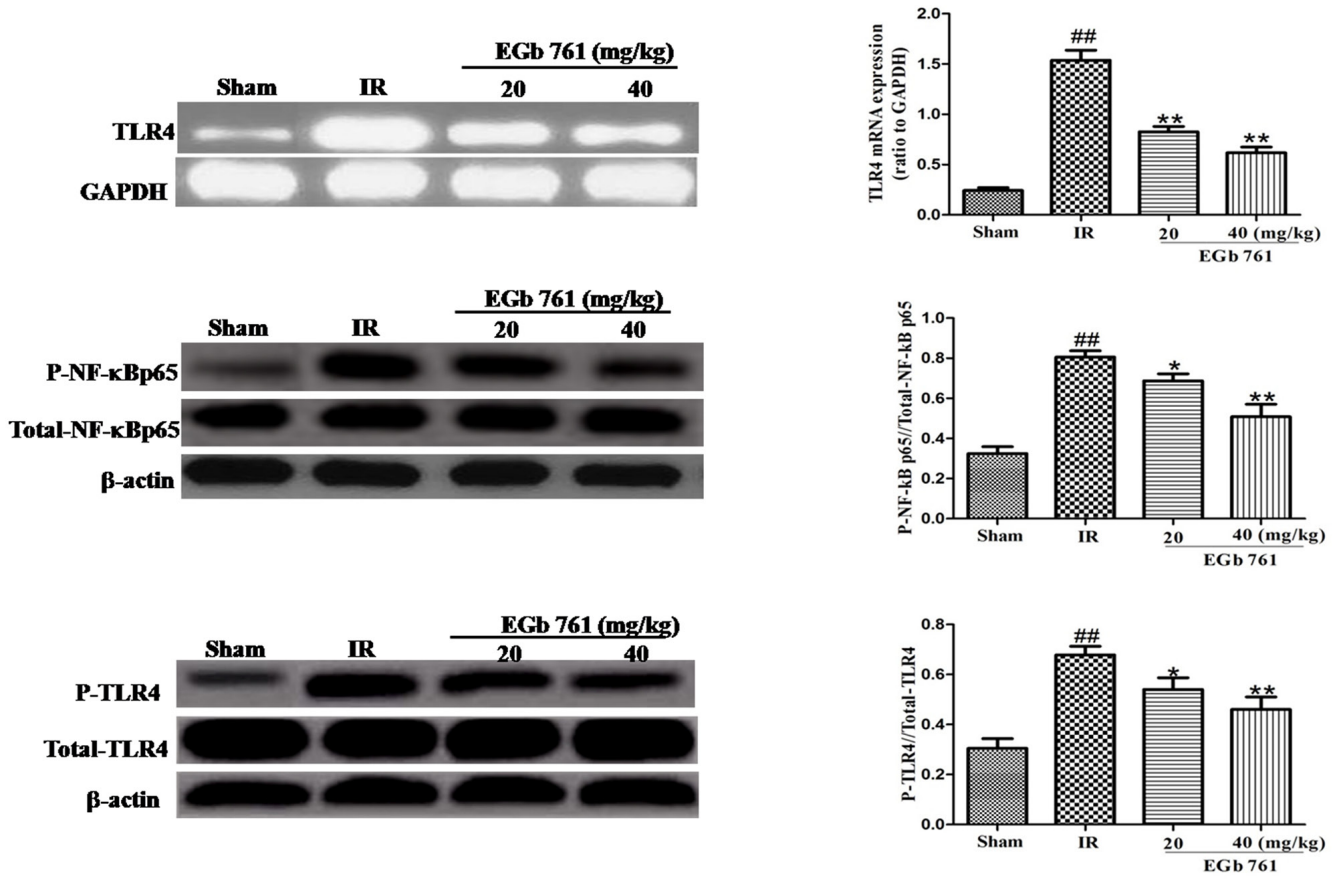
EGb 761 inhibited TLR-4/NF-κB-mediated inflammatory signalings in the myocardium tissue Values were expressed as means ± SDs. ## P <0.01 compared with sham group. *P < 0.05 and ** P < 0.01 compared with IR group (n=10).

In the present studys, the expression content of TLR4, p-TLR4, NF-κB p65 and p-NF-κB p65 were determined to find the effects of EGb 761 on TLR4 signaling pathway. Compared with the sham group rats, the expressions of p-TLR4 and p-NF-κB p65 both greatly raised a lot in I/R group. After administration of EGb 761 (20 and 40 mg/kg), the expressions of p-TLR4 and nuclear p-NF-κB p65 were reduced (Figure 9). These results indicated that the potential possessive reactions of EGb 761 on great I/R injury were on the basic of the inhibition of TLR4/NF-κB signaling pathway which may be a meaningful mechanism (Figure 9).

## DISCUSSION

In normal, healthy cells could availably scavenge free radicals by plenty of antioxidants. Nonetheless, the unusual formation of reactive oxygen species dramatically results in breaking the balance with the expanded demand on the antioxidant defense system in pathological conditions which is similar to ischemic–reperfusion injury (IRI). With the accumulation of reactive oxygen species, the endogenous antioxidants are depleted at the same time such as SOD, CAT and GPx. It is of possibility to restrict the oxidative stress and reduce the induced tissue damage in order to prevent or alleviate disease progression by facilitating the balance with less oxidative stress. Present studies have shown that the ischemic–reperfusion injury was in connection with the accrued oxidative stress which was proved by the depleted myocardial endogenous antioxidants of SOD, GSH-Px, GR and CAT. On the other side, the reactive oxygen species can be scavenged precisely by EGb 761. Also, this is supported by the reduced level contains of AST, LDH and CK-MB in EGb 761 group.

Most members of interleukin-1 family are the pro-inflammatory cytokines, which could stimulate the inflammatory and autoimmune response [25]. The results of this study indicated that the expression of inflammatory factors in IR group was up-regulated, which was closely related to the injury mechanism of involved cardiac muscle. The major active calcium of Ca^2+^–ATPase protein transport takes the responsibility for keeping the maintenance of normal calcium levels intracellularly in many cell types. The Na^+^/K^+^–ATPase (sodiumpump) behaves as heterodimer protein which crucially retains cell homeostasis. The present studies have indicated that the observed Na^+^/K^+^–ATPase and Ca^2+^–ATPase with decreased activity may be explained as the cardiovascular damage index which is induced by IR. TLR4 which in the immune reaction response is one of the pattern recognition receptors not only takes participates but also makes I/R injury contributions to the inflammatory response. It mainly plays its role by mediating the MyD88 dependent and independent pathways. Through the MyD88 dependent pathway, it could activate the NF-kB, cause its transfer in the nucleus and activate the transcription of cytokine to release the related inflammatory cytokines [26]. The production of cytokine could also play the role of positive feedback to further activate the NF-kB. Meanwhile, there was the certain regulation of negative feedback. When the activation of NF-kB was inhibited, the mediation of TLR-4 was reduced and the expression was blocked as well [26]. The administration of EGb 761 with 20 and 40 mg/kg could reduce both the quantity of p-TLR4 and nuclear expression of p-NF-κB p65. It may be an important mechanism to inhibit the signaling pathway of TLR4/NF-κB to potentially on I/R injury exert the positive effects of EGb 761.

In summary, the observations in this study extended the vision in rat model was demonstrated of the protective effects of EGb 761 against I/R injury for the first time which was related with the downregulation of TLR4/NF-κB and inhibition of inflammatory response.

## MATERIALS AND METHODS

### Main reagents and kits

TNF-α, IL-6 and IL-1β enzyme-linked immunosorbent assay (ELISA) kits were provided by Nanjing KeyGEN Biotech. CO., Ltd. (Nanjing, China). CK, AST, LDH, MDA, SOD, CAT, GSH-Px and GR assay kits were produced by Jiancheng Bioengineering Institute (Nanjing, China). Antibodies against TLR4, NF-κB p65, B-cell lymphoma-2 (Bcl-2) and B-cell lymphoma-2 associated X (Bax) were purchased from Cell Signaling Technology (Beverly, MA, USA). TLR4 primers were obtained from TaKaRa Biotechnology Co. (Dalian, China). (The TLR4 forward primer: up, CTATCATCAGTGTATCGGTG; down, CAGTCCTCATTCTGG CTC, the GAPDH forward primer: AATGCATCCTGCCACCACCAACTGC, reverse primer: GGAGGCCATGTAGTAGGCCATGAGGTC). The Chinese medicine of EGb 761 which contained 6% of terpene trilactones, 24% of flavonol glycosides and less than 5 ppm of ginkgolic acids was purchased from Jiangsu Shenlong Pharmaceutical Co., 110722, Yancheng, China.

### Animals

The Male Sprague-Dawley rats which were divided into four groups with 10 rats in each weighed 200–220 g and were provided by Shanghai Slac Laboratory Animal Co. LTD (Shanghai, China). The animals were fed individually under constant temperature (25 ± 1°C and humidity with a 12 h light/dark cycle and with a rodent standard diet with free access to water ad libitum. This study was performed in adherence with the National Institutes of Health Guidelines for the Use of Laboratory Animals.

### Chemical analysis of EGb 761

The multi-components of the ***G.*** *biloba* extract were characterized by UPLC-PDA-TOF/MS. An ACQUITY UPLC™ BEH C_18_ (2.1 ×100 mm I.D., 1.7 μm, Waters, Milford, USA) column was used for the analyses. The mobile phase composed of A (acetonitrile) and B (0.1% formic acid, v/v) with a gradient elution: 0-15 min, 5-95% A.

### Experimental protocol

All the male Sprague-Dawley rats were divided into four groups randomly: (1) Sham group dealt with saline vehicle but without I/R; (2) Ischemic reperfusion (IR) group given with saline alone; (3) I/R group given with EGb 761 of 20 mg/kg; (4) I/R group given with EGb 761 of 40 mg/kg. In the sham group and IR control group, the rats (10 rats in each) before IR operation were given saline by oral gavage for 15 d. In the two EGb 761 treatment groups, all the rats before IR operation were administered with EGb 761 (20 or 40 mg/kg) by oral gavage for 20 d. Rats were subjected to a coronary artery occlusion for 60 min followed by the reperfusion for 3 h. The snare encircling the coronary artery was used for occlusion by pulling up on the suture which was clamped with plastic tubes. Coronary artery reperfusion was restored by releasing the clamp. The sham groups were exposed to a time-matched normal perfusion without ischemia-reperfusion. For left ventricular (LV) pressure recordings, a water-filled small latex balloon by polythene cannula was inserted through left atrium into the left ventricle and was connected to the pressure transducer for the LV systolic and diastolic pressure measurements. The LV developed pressure (LVDP) was the difference between systolic and diastolic pressures. The LV end-diastolic pressure (LVEDP) was adjusted to 10mmHg at the beginning of the experiment and the LV pressures were differentiated to estimate the maximum rate of pressure development (+dP/dt_max_) and the maximum rate of LV pressure decay (-dP/dt_max_). At the end of reperfusion, blood samples were collected via carotid artery, then the left ventricle was obtained for determination of myocardial infarction size, and part of the anterior wall of the left ventricular myocardium near the cardiac apex was obtained for other analysis [27-29].

### Evaluation of myocardial infarction and injury

After 60 min of myocardial ischemia and 180 min of reperfusion, hearts were immediately removed, cleaned of blood with physiological saline via the aorta, injected with 1% TTC (triphenyltetrazolium chloride) until they became red, and snap frozen in liquid nitrogen for 20 min. Subsequently, the hearts were cut into cross-sectional pieces and fixed in 10% formaldehyde for 12 h. The TTC-stained sections were photographed. The percentage of infarct size is determined by weight [28, 29].

### Evaluation of antioxidant indices

SOD, CAT, GSH-Px, GR activities, MDA contents were used as indices of reactive oxygen species and membrane lipid peroxidation level. The content of MDA and activities of SOD, CAT, GSH-Px, GR were measured using commercial kits (JianCheng Bioengineering Institute, Nanjing, China) and analyzing with a spectrophotometer. Detailed manipulation process was performed according to the manufacturer’s instructions.

### Determination of release of AST, LDH, CK-MB into serum

Myocardial cellular damage was evaluated by measuring serum AST, LDH, and CK-MB levels. Serum AST and LDH activities were measured spectrophotometrically, and serum CK-MB was quantified using a commercial ELISA kit according to the manufacturer’s instructions.

### Determination of serum TNF-α, IL-6 and IL-1β

The levels of inflammatory cytokines, such as IL-6, IL-1β and TNF-α in the serum samples were quantified using specific ELISA kits for rat according to themanufacturers’ instructions.

### Estimation of myocardial injury markers

Activities of Na^+^–K^+^–ATPase and Ca^2+^–ATPase from cardiac tissues were determined using commercially available standard kits by RT-9600 Semi-automatic Biochemical Analyzer. All measurements were performed according to the kits manufacturers’ instructions, respectively [28].

### Histological examination of myocardium

Immediately after the sacrifice of the rats, the hearts were removed and fixed in 10% formalin solution. The heart tissue was processed for sectioning and staining by standard histological methods. Sections from the left ventricle were stained with hematoxylin and eosin (H&E) and examined by light microscopy at 200 × magnification.

### Reverse transcription-polymerase chain reaction analysis

The sample amount of total RNA (2 μg) was used from each sample. RNA was reverse transcribed using avian myeloblastosis virus reverse transcriptase with random hexamers for 50 min at 42 °C. The complementary DNA was amplified under the following cycle conditions: 4 min at 94 °C for one cycle, 30 cycles at 94 °C for 50 s, 54 °C for 30 s and 72 °C for 1 min and lastly a final extension for 5 min at 72 °C. The polymerase chain reaction products were analyzed by 2% agarose gel stained with ethidium bromide and ultraviolet irradiation. The semiquantitative measure for atrial natriuretic peptide and brain natriuretic peptide mRNA was expressed as a ratio to glyceraldehyde-3-phosphate dehydrogenase (GAPDH) mRNA.

### Western blot analysis

Nuclear proteins were extracted from heart tissue using nuclear and cytoplasm extraction kit according to the manufacturer’s instructions. The protein concentration was determined by the Bradford method. After boiling the samples for 5 min, the protein samples were fractionated by sodium dodecyl sulfate polyacrylamide gel electropheresis (SDS-PAGE) (10–12% polyacrylamide gels) and transferred to polyvinylidene fluoride membrane (Millipore, Bedford, MA, USA). The membranes were blocked with milk powder at room temperature for 2 h. The membranes were incubated with primary antibodies of TLR4, Bcl-2, Bax and p65 at room temperature for 2 h, following primary antibody incubations, membranes were incubated with horseradish peroxidaselinked secondary antibodies (antirabbit, antimouse or antigoat IgG) mouse or antigoat IgG.

### Statistical analysis

All values were expressed as the mean ± S.D. and analyzed by one-way analysis of variance (ANOVA) followed by Duncan's Multiple Range Test using SPSS version 13.0 software; a P-value of less than 0.05 was considered significant and P < 0.01 was considered to be statistically very significant.
